# Unlocking the potentials of community health workers for effective control of mpox outbreaks in Africa

**DOI:** 10.11604/pamj.supp.2025.50.1.45629

**Published:** 2025-03-02

**Authors:** Patrick Martial Nkamedjie Pete, Rodrigue Mabvouna Biguioh, Fai Karl Gwei Njuwa, Morenike Oluwatoyin Folayan, Christian Ngandu, Ngashi Ngongo, Nicaise Ndembi

**Affiliations:** 1 Africa Centres for Disease Control and Prevention (Africa CDC), Addis Ababa, Ethiopia,; 2 Alliance for International Medical Action, Dakar, Senegal, Nigeria,; 3 Homegrown Solutions for Health, Yaoundé, Cameroon, Nigeria,; 4 Department of Child Dental Health, Obafemi Awolowo University, Ile-Ife, Nigeria,; 5 COUSP, INSP, Kinshasa, Democratic Republic of Congo

**Keywords:** Mpox, community health workers, infection control, Africa

## Abstract

Since May 2022, multiple countries have reported clusters of monkeypox (mpox) virus infections. In response to the current upsurge of mpox in the Democratic Republic of the Congo and an increasing number of African countries, the Africa Centers for Disease Control and Prevention (Africa CDC) and World Health Organization (WHO) declared mpox as a Public Health Emergency of Continental Security and a Public Health Emergency of International Concern on the 13th and 14th of August, 2024, respectively. Although community health workers (CHWs) were instrumental in controlling the 2014/2015 Ebola outbreak across West Africa, little attention is given to their potential importance of CHWs in the prevention and control of large-scale mpox outbreaks. Community health workers (CHWs) are often considered trusted voices in the community and can be critical to help raise awareness on mpox and deliver preventive messages in a sensitive and culturally appropriate manner. Moreover, CHWs can be instrumental in addressing common misconceptions and false rumours that hinder healthcare-seeking behaviours. Another adaptive role played by well-trained and adequately equipped CHWs is surveillance through door-to-door active case finding of mpox cases, which is crucial in reducing dissemination at community level. Effectively tackling vaccine hesitancy is essential to ensure herd immunity and enhance mpox response. Community health workers (CHWs) can be seen as crucial resources in addressing myths and rumours, which are critical drivers of concerns on mpox vaccine safety and side effects; thus, bolstering vaccine acceptance and coverage. Community health workers (CHWs) play a central role in fostering partnership and collaboration between communities, health authorities and response actors for a harmonized approach to tackle mpox outbreaks.

## Perspective

Mpox is a zoonotic disease caused by the monkeypox virus (MPXV) which is an orthopoxvirus that belongs to the same genus as the smallpox virus. The virus was discovered in 1958, with the first human infection recorded in 1970 in the Democratic Republic of Congo (DRC). As of May 2022, clusters of monkeypox (mpox) virus infections have been reported across many countries [1]. In response to the current upsurge of mpox in DRC and an increasing number of countries in Africa, the Africa Centers for Disease Control and Prevention (Africa CDC) declared mpox as a Public Health Emergency of Continental Security (PHECS) on 13th August 2024. This was closely followed by a declaration from the World Health Organization (WHO) proclaiming the disease as a Public Health Emergency of International Concern (PHEIC) on 14th August 2024 [2]. The 3rd standing recommendations on mpox instructed by the Director-General of the World Health Organization (WHO) as per the new amendments of the International Health Regulations (2005), urged state parties to hardness community protection by strengthening capacity for risk communication and community engagement, tailoring public health and social measures to local contexts and promoting equity and building trust with local communities, especially for those most at risk [3].

Historically, clade I MPXV has been predominant, contributing to 95% of all reported cases. However, in 2017, a significant shift occurred during a widespread outbreak of MPXV Clade II in Nigeria, indicating sustained human-to-human transmission, including through the sexual route. In 2022-2023, a large global outbreak of mpox disease resulting from clade II monkeypox virus was reported, signaling the first instance of sustained transmission outside the African continent [4]. The disease cannot only be transmitted from animals to humans but can also be disseminated from human to human [5]. Evidence suggests that the natural reservoirs of mpox include animals such as squirrels, Gambian pouched rats, monkeys, dormice, nonhuman primates, and other species. Infections in humans occur by bite/scratch, close physical contact, and by consuming undercooked meat of infected animals. Human-to-human transmission occurs through large respiratory droplets, direct contact, and contaminated fomites. The rise in cases across the African continent is attributed to the decline of immunity resulting from the cessation of smallpox vaccination and increased infringement of forest areas for human activities [6]. Recommendations from WHO and CDC suggest that two existing smallpox vaccines, JYNNEOS and ACAM2000, possess an 85% efficacy against mpox [7]. Knowledge, cultural beliefs, past vaccination experiences, perceived vaccine benefits, and risks are factors that can impact people´s attitudes toward receiving the mpox vaccine, potentially resulting in vaccine hesitancy [8].

Although community health workers (CHWs) were instrumental in controlling the 2014/2015 Ebola outbreak across West Africa [9] and in implementing Côte d´Ivoire´s precautionary response to the epidemic in neighbouring Guinea and Liberia [10], little attention is given to the potential importance of CHWs in the prevention and control of large-scale mpox outbreaks. By leveraging their role in previous large-scale infectious disease outbreaks, CHWs can considerably improve the efficacy of the mpox response. CHWs are often considered trusted voices in the community and thus represent key assets for community mobilization and health information dissemination during outbreaks. They can raise awareness on mpox and deliver preventive messages in a sensitive and culturally appropriate manner. Moreover, CHWs can be instrumental in addressing common misconceptions and false rumours that hinder healthcare-seeking behaviours. Because mpox is of zoonotic origin, CHWs could also be used to develop and promote effective One Health messaging campaigns.

Glenton et al. argued that CHWs play key roles that contribute to strengthening health systems and reinforcing response to public health threats, including community mobilization, health promotion, provision of preventive services, provision of clinical services, epidemographic surveillance and record-keeping, creation of trust and social support [11]. Thus, another adaptive role CHWs can contribute to during mpox outbreaks is disease surveillance. CHWs often possess an in-depth understanding of local cultural practices and the reasons why community members may refuse to adhere to treatment protocols or report cases of diseases. Well-trained and adequately equipped CHWs are instrumental for door-to-door active case finding of mpox cases, which is crucial in reducing dissemination at the community level. Moreover, CHWs could also be considered an important component in setting up a robust mpox patient referral system from the community to health facilities, ensuring patients reach the health facilities where they receive appropriate treatment. Additional activities to reduce community transmission of mpox through contact tracing conducted by CHWs also warrant attention.

Remarkable strides have been made to develop mpox vaccines to mitigate mpox transmission and reduce associated mortality, especially for high-risk populations. However, vaccine hesitancy hampers progress towards future mpox vaccine uptake. Effectively tackling vaccine hesitancy is essential to ensure herd immunity and enhance mpox response. CHWs can be seen as crucial resources in addressing myths and rumours, which are critical drivers of concerns on mpox vaccine safety and side effects. Understanding the underlying reasons for vaccine hesitancy and fostering health education to improve vaccine knowledge and acceptance are paramount. Public health decision-makers and stakeholders should highly prioritize the roles of CHWs in leading mpox vaccine acceptance and hesitancy surveys at the community level.

In alignment with the Monrovia Call to Action, endorsed in Liberia, which urged the acceleration of the implementation of the Community Healthcare Worker initiative, Africa CDC called for the adoption of a unified one plan, one budget, and one M&E framework during the high-level ministerial meeting on community health workforce at the World Health Assembly in May 2023. This call for action was further reinforced in September 2023 by the launch of Joint Country Support Capacity Plans to support African Union (AU) member states in implementing a coordinated approach for planning community health systems interventions. In October 2023, the Community Health Delivery Partnership (CHDP) programme was launched to enhance the acceleration of community-level priorities in-country through primary health care. A collective approach to financing community health in Africa was announced at the Reaching the Last Mile Health Forum in December 2023, securing a $900M commitment to strengthen community health systems, 74% of which was allocated to the African continent.

For successful mpox outbreak response strategies, priority must be given to community expectations and needs. Implementing and Institutional organizations must seek community ownership, engagement, and buy-in to ensure adherence to preventive measures, commitment to the overall outbreak response strategy, and facilitation of empowerment. Designing and implementing successful response strategies against mpox outbreak requires collaborative and concerted efforts between policymakers, the Ministry of Health, and local communities. Community health workers (CHWs) play a central role in fostering partnership and collaboration between communities, health authorities, and response actors for a harmonized approach to tackle outbreaks, resulting in improved mobilization of community resources and better management of community needs.

**Disclaimer:** this content is solely the responsibility of the authors and does not necessarily reflect the views of their affiliated institutions.
